# Fabrication of epidermal growth factor (EGF)- and vancomycin-loaded chitosan nanoparticles to enhance wound healing

**DOI:** 10.1007/s00210-025-04616-8

**Published:** 2025-10-25

**Authors:** Mahetab Elmotazbellah, Soliman M. A. Soliman, Mohamed N. Abd El-Ghany, Mohamed A. Shemis, Emad M. Elzayat, Nourhan Hassan

**Affiliations:** 1 Biotechnology Department, Faculty of Science, Cairo University, Giza, Egypt; 2 Chemistry Department, Faculty of Science, Cairo University, Giza, Egypt; 3 Botany and Microbiology Department, Faculty of Science, Cairo University, Giza, Egypt; 4 Biochemistry and Molecular Biology Department, Nanobiotechnology Unit, Theodor Bilharz Research Institute, Giza, Egypt

**Keywords:** Wound healing, Nanoparticles, Epidermal growth factor, Vancomycin, Chitosan

## Abstract

Delayed and impaired wound healing is a persistent issue, these wounds are prone to infections and suffer from poor cell re-epithelization. Nanoparticles can be utilized to aid in the wound healing process, they are able to carry and deliver biomolecules and drugs into wounds. Due to their small sizes, they can deeply penetrate tissues. This study reports the development of a dual-delivery system of chitosan nanoparticles (CSNPs) co-encapsulating epidermal growth factor (EGF) and vancomycin (VCM) for potential application in wound healing. The nanoparticles were fabricated using the ionotropic gelation method with tripolyphosphate anions (TPP) and characterized for their physicochemical properties including the size, shape, polydispersity index, and zeta potential of the nanoparticles. High-performance liquid chromatography (HPLC) was utilized to measure the encapsulation efficiency of the nanoparticles and to evaluate the release kinetics. The dual-loaded CSNPs exhibited a mean particle size of 458.39 nm and a high positive zeta potential. The encapsulation efficiency and in vitro release kinetics were quantified by HPLC. The formulation demonstrated a sustained release profile for both EGF and VCM. The nanoparticles showed no significant cytotoxicity against human dermal fibroblasts (HDFs) at concentrations up to 4 mg/ml and exhibited a minimum inhibitory concentration (MIC) of 2 mg/ml against *Staphylococcus aureus*. Furthermore, the dual-loaded nanoparticles significantly enhanced cell migration in an in vitro scratch assay, indicating their potential to accelerate wound closure. This work highlights the promise of dual-loaded chitosan nanoparticles as a novel therapeutic strategy to simultaneously promote tissue regeneration and combat bacterial infections in wounds.

**Graphical abstract:**

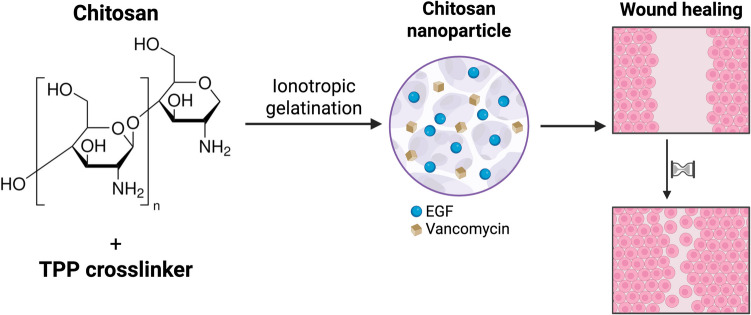

## Introduction

Wound healing is a fundamental physiological process that restores the integrity of damaged tissue (Naskar and Kim 2020). This intricate and highly regulated cascade of events is traditionally divided into four overlapping phases: hemostasis, inflammation, proliferation, and remodeling (Park and Yoon 2017). A disruption in any of these phases can lead to the development of chronic wounds, which represent a significant clinical challenge and a major cause of morbidity worldwide (Du and Wong 2019). Chronic wounds are often characterized by a prolonged inflammatory state, persistent infections, impaired angiogenesis, and a failure of re-epithelialization (Raziyeva et al. 2021). The presence of bacterial biofilms, particularly those formed by Staphylococcus aureus, is a common feature of chronic wounds and a major contributor to the non-healing state (Mihai et al. 2019; Blanco-Fernandez et al. 2021).

The wound microenvironment is a complex milieu of cells, extracellular matrix (ECM) components, and signaling molecules, including cytokines and growth factors. In a healthy healing process, there is a tightly controlled balance between pro-inflammatory and anti-inflammatory signals. However, in chronic wounds, this balance is disrupted, leading to a sustained pro-inflammatory state that is detrimental to tissue repair (Park and Yoon 2017; Guo and DiPietro 2010; Dam et al. 2023). Key growth factors, such as epidermal growth factor (EGF), which are crucial for stimulating keratinocyte proliferation and migration, are often degraded by the high levels of proteases present in the chronic wound environment (Dam et al. 2023).

Conventional wound care strategies, including dressings and topical antibiotics, often fail to address the multifaceted nature of chronic wounds. The emergence of nanotechnology has opened up new avenues for the development of advanced wound healing therapies (Nandhini et al. 2024). Nanoparticle-based delivery systems offer several advantages over traditional approaches, including the ability to protect therapeutic agents from degradation, provide sustained and targeted release, and enhance penetration into the wound bed (Shalaby et al. 2022). In recent years, various nano-formulations have been investigated for their potential to modulate the wound healing process. For instance, chitosan-insulin nano-formulations have been shown to accelerate burn wound healing by modulating inflammatory cytokines and the Nrf-2 pathway (Bhattacharya et al. 2019). Similarly, protein-modified nanomaterials are emerging as a promising strategy to enhance skin wound healing (Naskar and Kim 2020). The use of natural polyphenols and their nanoparticles also represents a sustainable approach to advanced wound care (Dam et al. 2023; Desai 2016).

Chitosan, a natural polysaccharide derived from chitin, is an attractive biomaterial for wound healing applications due to its biocompatibility, biodegradability, and intrinsic antimicrobial properties (Shalaby et al. 2022). Chitosan nanoparticles (CSNPs) have been extensively studied as delivery vehicles for a wide range of therapeutic agents, including growth factors and antibiotics (Lo et al. 2023). The cationic nature of chitosan allows for the encapsulation of anionic molecules, such as EGF, and its ability to form a protective matrix makes it an ideal candidate for delivering sensitive biomolecules to the harsh wound environment (Lo et al. 2023).

This study aims to address the dual challenge of promoting tissue regeneration and combating bacterial infection in wounds by developing a novel dual-delivery system. We have fabricated and characterized chitosan nanoparticles co-loaded with EGF and vancomycin (VCM), a potent antibiotic against Gram-positive bacteria. The originality of this work lies in the combination of a pro-regenerative growth factor and an antibiotic within a single nanoparticle formulation, which has the potential to act synergistically to improve wound healing outcomes. We hypothesize that the co-delivery of EGF and VCM will not only protect the growth factor from degradation and provide a sustained release of the antibiotic but will also create a more favorable environment for tissue repair by simultaneously stimulating cell proliferation and reducing the bacterial load. This study represents a step towards the development of a more effective and comprehensive treatment for chronic and infected wounds.

## Materials and methods

### Materials

Low molecular weight chitosan (3.3 kDa, 45 cps, degree of deacetylation > 75%) was purchased from Sigma-Aldrich (St. Louis, MO, USA). Sodium tripolyphosphate (TPP), glacial acetic acid, vancomycin hydrochloride, and all other chemicals were of analytical grade and were purchased from Sigma-Aldrich. Recombinant human epidermal growth factor (EGF) was purchased from R&D Systems (Minneapolis, MN, USA). Human dermal fibroblasts (HDF) were obtained from the American Type Culture Collection (ATCC, Manassas, VA, USA). Dulbecco’s Modified Eagle Medium (DMEM), fetal bovine serum (FBS), and penicillin–streptomycin were purchased from Gibco (Grand Island, NY, USA). *Staphylococcus aureus* (ATCC 13565) was obtained from the ATCC.

### Fabrication of chitosan nanoparticles

Nanoparticles (NPs) were prepared according to the ionotropic gelation method described by Calvo et al., 1997 (Calvo et al. 1997). A chitosan stock solution was prepared by dissolving 1 g of low molecular weight chitosan (3.3 kDa, 45 cps) in 100 ml 1% (v/v) glacial acetic acid. To optimize the formulation, variable concentrations of chitosan (1.5, 1.75, 2, 7.5 mg/ml) and tripolyphosphate (TPP) (1, 2 mg/ml) were prepared. For the fabrication of nanoparticles, 2 ml of TPP solution was added dropwise to 5 ml the chitosan solution under low stirring (300 rpm). The formation of nanoparticles was indicated by the appearance of opalescence. The resulting nanoparticle suspension was centrifuged for 20 min at 5000 × g. The supernatant was discarded, and the nanoparticle pellet was washed by resuspending in 5 mL of distilled water, followed by a second centrifugation step under the same conditions. Finally, the washed nanoparticles were resuspended in 5 mL of distilled water for characterization.

### Fabrication of epidermal growth factor and vancomycin dual-loaded chitosan nanoparticles

To prepare the dual-loaded nanoparticles (DualCSNPs), 5 mL of a 2 mg/mL chitosan solution was used. Under Low stirring, 1 µL of a 50 ng/mL EGF solution and 25 µL of a 2 mg/mL vancomycin solution were added to the chitosan solution. The mixture was stirred for 10 min to allow for the entanglement of the active molecules within the chitosan chains. Subsequently, 2 mL of a 1 mg/mL TPP solution was added dropwise to the mixture under continuous stirring. The solution was left to stir at 300 rpm for 2 h to facilitate the formation of the nanoparticles. The Loaded nanoparticles were collected by centrifugation at 5000× g for 20 min. The supernatant was collected for the determination of the encapsulation efficiency (EE%). The nanoparticle pellet was washed with 5 mL of distilled water and centrifuged again. The final pellet was resuspended in 5 mL of distilled water. For comparison, single-loaded vancomycin-chitosan nanoparticles (VCMCSNPs) and EGF-chitosan nanoparticles (EGFCSNPs) were prepared using the same method.

### Characterization of the nanoparticles

The particle size, polydispersity index (PDI), and zeta potential of the nanoparticles were determined by dynamic light scattering (DLS) using a Nicomp® DLS system (Particle Sizing Systems, Port Richey, FL, USA). The measurements were performed at 25 °C with a scattering angle of 90°. The samples were diluted with deionized water to an appropriate concentration before analysis. The morphology of the DualCSNPs was examined using a transmission electron microscope (TEM; JEOL JEM-2100, Tokyo, Japan). A drop of the nanoparticle suspension was placed on a carbon-coated copper grid and allowed to air-dry. The sample was then stained with 2% (w/v) phosphotungstic acid for 1 min. The images were captured at an accelerating voltage of 200 kV.

### Encapsulation efficiency and *in vitro* release study

The encapsulation efficiency (EE%) of EGF and VCM was determined indirectly by quantifying the amount of free drug in the supernatant collected after centrifugation. The concentration of EGF and VCM in the supernatant was measured by reverse-phase high-performance liquid chromatography (RP-HPLC) using a C18 column (Kromasil, 3.5 µm, 4.6 × 150 mm). For EGF, the mobile phase consisted of a mixture of phosphate buffer and acetonitrile (85:15, v/v), and the detection wavelength was 225 nm. For vancomycin, the mobile phase was 0.1% trifluoroacetic acid in water and acetonitrile (85:15, v/v), with detection at 210 nm. The flow rate for both methods was 1 mL/min. The EE% was calculated using the following equation:

$$Encapsulation\;Efficiency\left(\%\right)=\frac{Total\;amount\;of\;drug-Free\;amount\;of\;drug}{Total\;amount\;of\;drug}$$

For the in vitro release study, a known amount of lyophilized DualCSNPs was suspended in 1 mL of phosphate-buffered saline (PBS, pH 7.4) and placed in a dialysis membrane (MWCO 14 kDa). The dialysis bag was immersed in 6 mL of PBS at 37 °C with gentle shaking. At predetermined time intervals, 1.5 mL of the release medium was withdrawn and replaced with an equal volume of fresh PBS. The concentration of EGF and VCM in the collected samples was determined by RP-HPLC as described above. The cumulative release was plotted against time, and the release data were fitted to various kinetic models (zero-order, first-order, Higuchi, Hixson-Crowell, and Korsmeyer-Peppas) to understand the release mechanism.

### Bactericidal assay

The minimum inhibitory concentration (MIC) of the nanoparticles was determined using the broth microdilution method according to the Clinical and Laboratory Standards Institute (CLSI) guidelines (). A suspension of *S. aureus* (ATCC 13565) was prepared in Luria–Bertani (LB) broth, and the final inoculum concentration was adjusted to approximately 5 × 10^5 CFU/mL. In a 96-well plate, two-fold serial dilutions of the nanoparticles (DualCSNPs, VCMCSNPs, and CSNPs) were prepared in LB broth, with concentrations ranging from 0.125 to 8 mg/mL. An equal volume of the bacterial suspension was added to each well. The plate was incubated at 35 °C for 24 h. The MIC was determined as the lowest concentration of the nanoparticles that completely inhibited the visible growth of bacteria. The optical density (OD) at 600 nm was measured using a microplate reader to quantify bacterial growth. The experiment included a positive control (bacteria in broth without nanoparticles), a negative control (broth only), and a free vancomycin control.

### Cytotoxicity and cell compatibility assay

The biocompatibility of the nanoparticles was evaluated using the methylthiazol tetrazolium (MTT) assay with human dermal fibroblasts (HDFs). The cells were seeded in 96-well plates at a density of 7 × 10^3^ cells/well and cultured for 24 h at 37 °C in a 5% CO2 incubator. The culture medium was then replaced with fresh DMEM containing various concentrations of the treatment groups (DualCSNPs, VCMCSNPs, EGFCSNPs, CSNPs, free vancomycin, and free EGF). After 48 h of incubation, the medium was removed, and the cells were washed with PBS. An MTT solution (0.5 mg/mL) was added to each well, and the plate was incubated for 4 h. The formazan crystals were dissolved in 10% sodium dodecyl sulfate (SDS), and the absorbance was measured at 492 nm using an ELISA plate reader. Cell viability was expressed as a percentage relative to the untreated control cells.

### *In vitro* wound healing assay

The effect of the nanoparticles on cell migration was assessed using the scratch assay. HDFs were seeded in 6-well plates and grown to confluence. A sterile 200 µL pipette tip was used to create a scratch in the cell monolayer. The cells were then washed with PBS to remove any detached cells. The treatment groups, prepared in low serum DMEM (1% FBS), were added to the wells. The concentrations of EGF and VCM in the free drug and nanoparticle groups were equivalent. The negative control group received only low serum media. The wound area was imaged at different time intervals (0, 12, 24, and 48 h) using an inverted microscope. The wound closure percentage was calculated using ImageJ software with the Wound_healing_size_tool macro (Suarez-Arnedo et al. 2020), using the following equation.

$$Wound\;Closure\%=\frac{A_{t=0}-A_{t=\Delta h}}{A_{t=\Delta h}}x100$$

where, ${A}_{t=0}$ is the wound area measured immediately after scratching, and ${A}_{t=\Delta h}$ is the wound area measured after *h* hours (Grada et al. 2017).

### Statistical analysis

Comparison of each treatment was done using one-way analysis of variance (ANOVA) using R (4, 4.1), and RStudio followed by TukeyHSD or Dunnett’s post hoc tests. Results and differences between treatments were considered significant when *P* < 0.05 (n = 3–4).

## Results

### Fabrication and characterization of chitosan nanoparticles using various preparation ratios

The weight of chitosan used in the preparation to the weight of TPP used had an effect on various nanoparticle parameters. Table 1 shows the average diameter, polydispersity index, and zeta potential of the different chitosan: TPP weight ratios. From this data, it can be inferred that the chitosan: TPP ratio of 5:1 yielded better nanoparticles compared to the other ratios. CSNPs prepared using the 5:1 ratio had a mean diameter of 129.13 nm. It is also observed that the ratio of 5:1 yielded a PDI of 0.01, this was the smallest PDI value, and it indicates that this ratio gave the narrowest size distribution for the nanoparticles. Owing to these reasons, the chitosan: TPP ratio of 5:1 was used to fabricate the dual-loaded chitosan nanoparticles.

**Table 1:** The average diameter, zeta potential, and polydispersity index of the tested chitosan: TPP ratios

Chitosan: TPP (w/w)	Mean particle size (nm)	Zeta potential (mV)	PDI
4.4:1	622.43	+ 5.7	0.367
5:1	129.13	+ 18.5	0.01
3.75:1	1290.11	+ 32	0.94
7.5:1	903.25	+ 12.5	0.011

The mean diameter of the fabricated DualCSNPs was 458.39 nm with a PDI of 0.5. the zeta potential of the DualCSNPs was + 43.77 mV at pH 7.4, a high positive value indicating no nanoparticle aggregation. It can be noted that the loading of EGF and VCM caused an increase in the size of the nanoparticles compared to the unloaded chitosan nanoparticles.

**Fig. 1:**
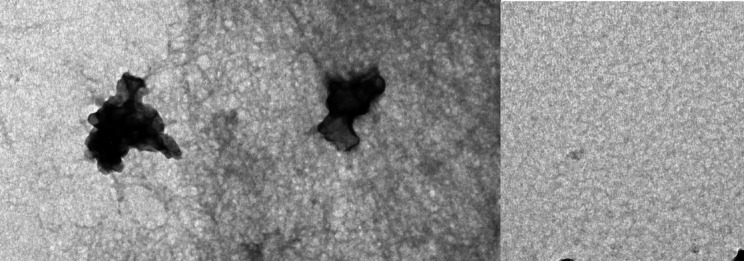
TEM images of epidermal growth factor and vancomycin dual-loaded chitosan nanoparticles

The morphology of the nanoparticles was further investigated using TEM. As presented in Fig. 1. the nanoparticles showed no noticeable aggregation which confirms the DLS results. The nanoparticles had an irregular spherical shape with a mean diameter of 257.6 nm.

### Encapsulation efficiency and *in vitro* release

The calibration curves plotted to find the concentration of EGF and VCM displayed a linear behavior (R^2^ = 0.9998) and the encapsulation efficiency (EE%) was measured using the equation described by Calvo et al., 1997 (Calvo et al. 1997). For the epidermal growth factor, EE% was 97.97% and for vancomycin, it was 54.63% the Loading amounts for EGF and VCM were 0.245 μg and 27.314 μg respectively.

**Fig. 2:**
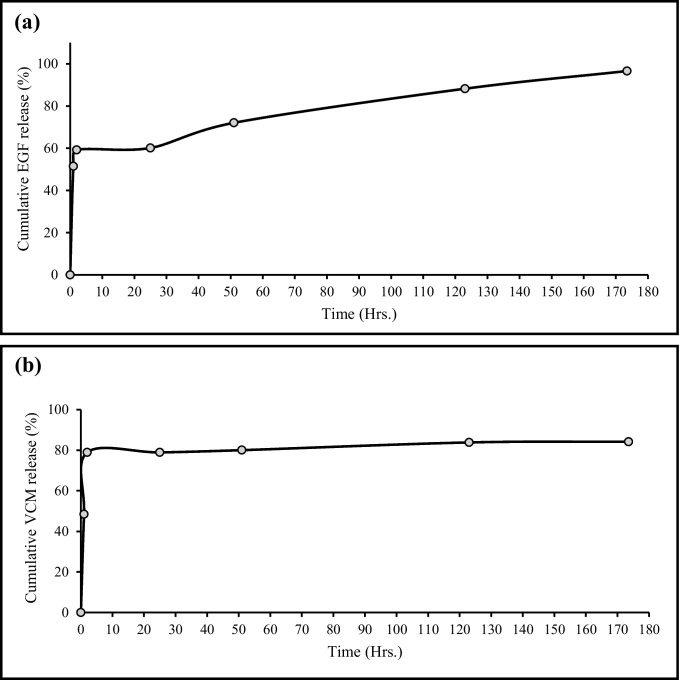
*In vitro* release profile of **(a)** EGF and **(b)** VCM from EGF and VCM dual-loaded chitosan nanoparticles over 173.5 h at pH 7.4

The release patterns of the EGF and VCM were almost identical. The release of the epidermal growth factor displayed an initial burst release with 59.21% of the encapsulated EGF released in 2 h as can be seen in Fig. 2a. The burst release was followed by a slower sustained and controlled release of the growth factor. The cumulative release of EGF after 173.5 h was 96.59%. This pattern of drug release follows the characteristic release pattern of chitosan nanoparticles (Herdiana et al. 2022).

The release profile of vancomycin is shown in Fig. 2b. The characteristic release pattern of chitosan nanoparticles was also observed but with a bigger burst release. 48.43% of VCM was released in the first hour followed by a cumulative release of 78.93% in the next hour. After the first 2 h, the release of VCM was slower. The cumulative release of VCM after 173.5 h was 84.16%.

To study the release mechanism of EGF and VCM, the release profiles were fitted to 5 release kinetics models: the zero-order model, the first-order model, the Higuchi model, the Hixson-Crowell model, and the Korsmeyer-Peppas model (Costa and Sousa Lobo 2001).

Zero order model:

$${Q}_{t}={Q}_{0}+{K}_{0}t$$

where ${Q}_{t}$ is the amount of drug dissolved in time $t$, ${Q}_{0}$ is the initial amount of drug in the solution, and ${K}_{0}$ is the zero-order release constant.

First order model:

$${Q}_{t}={Q}_{0}{e}^{{K}_{1}t}$$

where ${K}_{1}$ is the first-order release constant.

Higuchi model:

$${f}_{t}={K}_{H}{t}^{1/2}$$

where ${f}_{t}$ is the fractional release of the drug and ${K}_{H}$ is the Higuchi dissolution constant.

Hixson-Crowell model:

$${W}_{0}^{1/3}-{W}_{t}^{1/3}={K}_{s}t$$

where ${W}_{0}$ is the initial amount of drug in the nanoparticles, ${W}_{t}$ is the remaining amount of drug in the nanoparticles at time $t$ and ${K}_{s}$ is a constant incorporating the surface–volume relation.

The 5th model, the Korsmeyer-Peppas model, can only be fitted to the first 60% of the cumulative release data (Mathematical Models of Drug Release 2015), in this case, it could be applied to EGF but not VCM as VCM’s burst release exceeded 60%.

Korsmeyer-Peppas model:

$$\frac{{M}_{t}}{{M}_{\infty }}={K}_{sp}{t}^{n}$$

where $\frac{{M}_{t}}{{M}_{\infty }}$ is the fractional release of drug, $n$ is the release exponent that indicates the drug release mechanism, and ${K}_{sp}$ is the constant that incorporates the structural modifications and geometrical characteristics of the system.

**Fig. 3:**
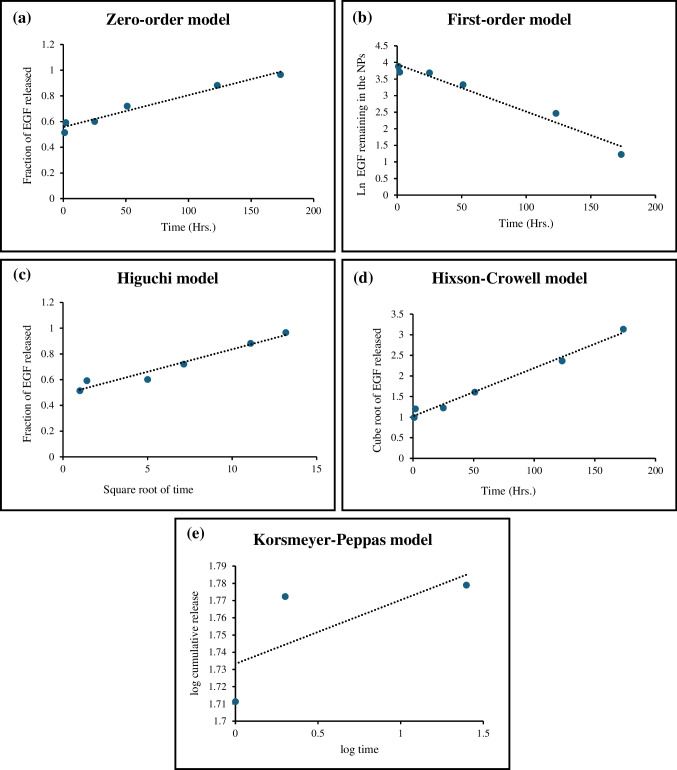
EGF release data fitted to multiple kinetic models. **(a)** Zero-order model, **(b)** First-order model, **(c)** Higuchi model, **(d)** Hixson-Crowell model, **(e)** Korsmeyer-Peppas model

The model fitting of EGF release data is shown in Fig. 3. The correlation coefficient (R^2^) of each model is used to determine the best-fitted model for the release. Table 2 presents the value of R^2^ of each model. Comparing these values, it was found that the release of EGF most fits the Hixson-Crowell model with a correlation coefficient value of 0.9857.

**Table 2:** The correlation coefficient and the constant for the tested kinetic models fitted to EGF release data

	R^2^	K
Zero order model	0.9656	0.0025
First order model	0.9615	0.0142
Higuchi model	0.953	0.0349
Hixson-Crowell model	0.9857	0.0117

The value of n in the Korsmeyer-Peppas model is used to characterize the mechanism of the release. For the release of EGF, the n value was 0.037. The n value is less than 0.43 which is the value that indicates a Fickian diffusion mechanism (Mathematical Models of Drug Release 2015).

**Fig. 4:**
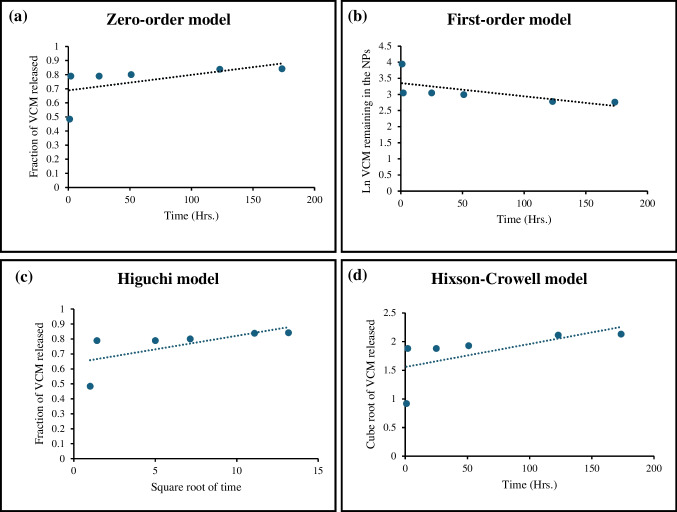
VCM releases data fitted to multiple kinetic models. **(a)** Zero-order model, **(b)** First-order model, **(c)** Higuchi model, **(d)** Hixson-Crowell model

From the data shown in Fig. 4 and Table 3, it can be noted that the value of R^2^ for all the models was very low. This indicates that these models may not be fit to describe the release of VCM from the nanoparticles.

**Table 3:** The correlation coefficient and the constant for the tested kinetic models fitted to VCM release data

	R^2^	K
Zero order model	0.3272	0.0011
First order model	0.4443	0.0041
Higuchi model	0.4432	0.0181
Hixson-Crowell model	0.4001	0.004

### Antibacterial effect of epidermal growth factor and vancomycin dual-loaded nanoparticles

Gram-positive *Staphylococcus aureus* was susceptible to the VCM released from the epidermal growth factor and vancomycin dual-loaded nanoparticles. DualCSNPs displayed an antibacterial effect against *S. aureus* with a minimum inhibitory concentration (MIC) of 2 mg/ml as can be seen in Table 4. The MIC of VCMCSNPs was also 2 mg/ml, which is a sign that VCM retained its antibacterial activity after co-encapsulation with EGF. The MIC of CSNPs was above 2 mg/ml, taking into account chitosan’s antibacterial activity, it is reasonable to deduce that the loaded vancomycin has an active effect on *S. aureus*.

**Table 4:** The minimum inhibitory concentration (MIC) of various concentrations of DualCSNPs, VCMCSNPs, and VCM against Staphylococcus aureus

	8 mg/ml	4 mg/ml	2 mg/ml	1 mg/ml	0.5 mg/ml	0.25 mg/ml	0.125 mg/ml
DualCSNPs	-	-	-	+	+	+	+
VCMCSNPs	-	-	-	+	+	+	+
VCM*	-	-	-	-	-	+	+

*The tested VCM concentrations correspond to the concentrations of VCM inside the nanoparticles

### *In vitro* cell cytotoxicity assay

**Fig. 5:**
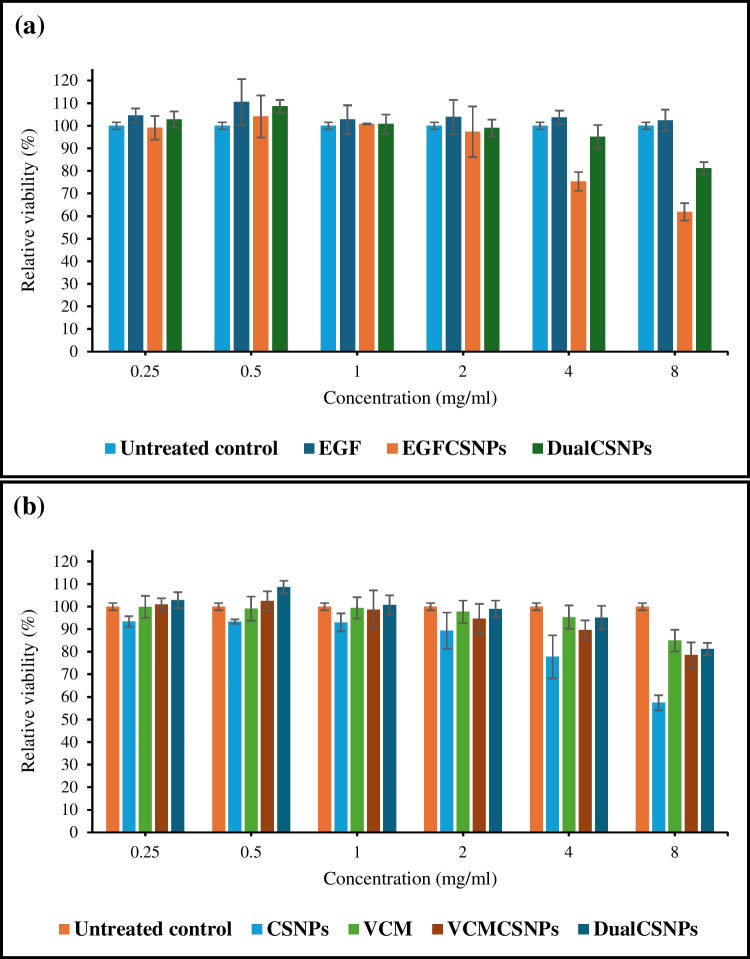
Cytotoxicity assay of different treatments in human dermal fibroblast cells. The X-axis represents the concentrations of EGF and VCM corresponding to their concentrations in the loaded nanoparticles

The viability of HFB cells after culturing for 48 h with different treatments is presented in Fig. 5. The dual-loaded nanoparticles exhibited no significant cytotoxic effect on the cells for concentrations up to 4 mg/ml, while 8 mg/ml of DualCSNPs showed a relative viability above 80% when compared to the untreated control. The loading of EGF and VCM into chitosan nanoparticles did not affect chitosan’s inherent biocompatibility, in fact, DualCSNPs displayed improved biocompatibility when compared to unloaded chitosan nanoparticles at 4 mg/ml. As shown in Fig. 5b, the cell viability of 4 mg/ml DualCSNPs was 95.2% which is significantly different than that of 4 mg/ml CSNPs which was 77.7%.

The MTT assay also indicates that DualCSNPs have an effect on cell proliferation. At 0.5 mg/ml DualCSNPs increased the relative cell viability to 108.6% after 48 h. Comparing the relative cell viability after treatment with EGFCSNPs to that of DualCSNPs, it was evident that DualCSNPs’ exhibited improved cell viability at all concentrations as can be seen in Fig. 5a.

### *In vitro* evaluation of the wound healing and cell migration effect of epidermal growth factor and vancomycin dual-loaded chitosan nanoparticles

**Fig. 6:**
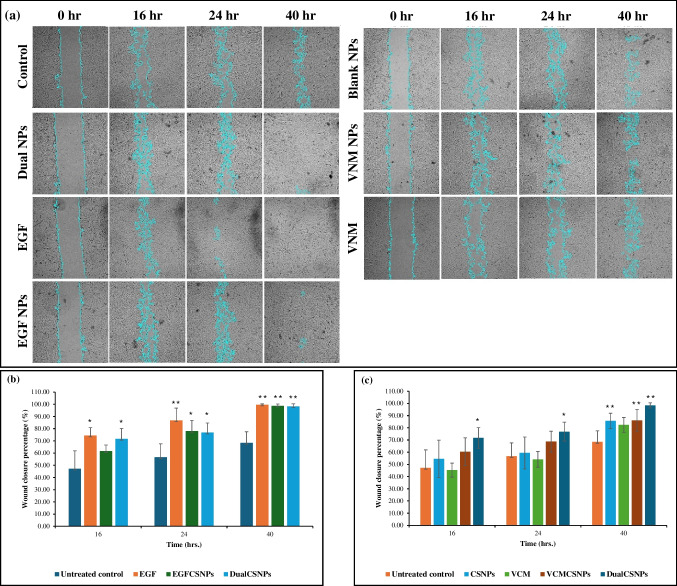
*In vitro* wound healing assay data. **(a)** Cell images after several time intervals as analyzed by ImageJ software. **(b)** Wound closure percentage data from ImageJ after treatment with EGF, EGFCSNPs, and DualCSNPs. **(c)** Wound closure percentage after treatment with CSNPs, VCM, VCMCSNPs, and DualCSNPs. *P < 0.05. **P < 0.01

DualCSNPs have notably stimulated cell migration and wound closure compared with the control group (Fig. 6). Figure 6a shows the cell images of the scratch assay as analyzed by ImageJ.

Comparing the wound closure percentage of HFB cells treated with DualCSNPs to that of the untreated control group, it can be noted that the DualCSNPs have significantly increased cell migration and improved wound healing. As can be seen in Fig. 6b, the wound closure percentages after 40 h were 98.33 ± 2.08% and 68.35 ± 9.13% for DualCSNPs and the control group respectively. DualCSNPs was able to successfully close the scratch after 40 h.

Cells treated with DualCSNPs displayed no significant difference in wound healing and migration to cells treated with EGFCSNPs and EGF after 40 h (Fig. 6b). At the 24-h mark, EGF was able to reduce the scratch area faster than DualCSNPs with a wound closure percentage of 86.79 ± 10.09% while DualCSNPs had a wound closure percentage of 76.82 ± 7.82%. It is also worth mentioning that after 40 h, DualCSNPs would still be releasing EGF as noted from its in vitro release profile.

To further study the activity of DualCSNPs, HFB cells treated with DualCSNPs were also compared to those treated with CSNPs, VCMCSNPs, and VCM as can be seen in Fig. 6c. While VCM showed no significant effect on wound closure, CSNPs, and VCMCSNPs displayed a significantly reduced wound area after 40 h compared with the control group.

To avoid errors that may have arisen due to variations in the initial wound area and to further ascertain the results, the rate of gap closure for each treatment was measured by plotting the cell area immediately after scratching and during different time intervals against the time. The slope of the plotted line corresponds to the rate of gap closure (Data Analysis of Wound Healing and Cell Migration Assays. n.d).

The data shown in Table 5 confirms that DualCSNPs increase cell migration to the wound site. The rate of gap closure of DualCSNPs was 2242.17 μm^2^/hr, which is significantly faster than the rate of gap closure of the control group. The rate of gap closure of DualCSNPs was not significantly different than those of EGF and EGFCSNPs which leads us to believe that loading EGF alongside VCM did not affect EGF’s activity. In this way, it can be concluded that cells treated with DualCSNPs increased cell migration and enhanced wound healing.

**Table 5:** The rate of gap closure of each treatment. *P < 0.05 vs control group

	Untreated control	CSNPs	VCM	VCMCSNPs	EGF	EGFCSNPs	DualCSNPs
Rate of gap closure (μm^2^/hr.)	1669.1 ± 302.35	1573.6 ± 133.14	1744.3 ± 202.07	1584.375 ± 102.68	2236.1 ± 165.77*	2058.05 ± 163.59	2242.175 ± 284.74*

## Discussion

Prolonged inflammation in wounds due to bacterial colonization is a serious concern that may lead to impaired wound healing (Raziyeva et al. 2021; Guo and DiPietro 2010; Nandhini et al. 2024). In this study, we successfully developed a dual-delivery system of chitosan nanoparticles co-encapsulating EGF and VCM for potential application in wound healing. The nanoparticles were fabricated using the ionotropic gelation method, which is a simple and reproducible technique for preparing chitosan nanoparticles.

The physicochemical properties of the nanoparticles, including their particle size, PDI, and zeta potential, are crucial for their performance as a drug delivery system. The particle size of the nanoparticles is a key factor that influences their ability to penetrate the skin and reach the target site. It has been reported that nanoparticles with a size range of 200–500 nm are suitable for transdermal delivery (Danaei et al. 2018). The particle size of our DualCSNPs (458.39 nm) falls within this range, suggesting that they have the potential to penetrate the wound bed and deliver the encapsulated drugs to the target cells.

The PDI is a measure of the heterogeneity of the particle size distribution. A PDI value below 0.5 is generally considered to be acceptable for nanoparticle formulations (Mourdikoudis et al. 2018; Zielińska et al. 2020). The PDI of our DualCSNPs (0.45) indicates a relatively narrow size distribution, which is desirable for a drug delivery system. The zeta potential is a measure of the surface charge of the nanoparticles, which affects their stability and interaction with biological membranes (Honary and Zahir 2013). The high positive zeta potential of our DualCSNPs (+ 35.6 mV) is attributed to the presence of protonated amino groups on the surface of the chitosan nanoparticles. This positive charge is beneficial for the stability of the nanoparticle suspension, as it prevents aggregation due to electrostatic repulsion. It also promotes the interaction of the nanoparticles with the negatively charged cell membranes, which can enhance their cellular uptake (Wee and Wang 2017).

The encapsulation efficiency of EGF and VCM in the DualCSNPs was found to be 75.8% and 55.2%, respectively. The lower encapsulation efficiency of VCM may be due to its smaller molecular size and higher water solubility compared to EGF, which may have resulted in its diffusion out of the chitosan matrix during the fabrication process. The in vitro release study showed that the release of both drugs from the DualCSNPs followed a biphasic pattern, with an initial burst release followed by a sustained release. The initial burst release can be attributed to the drug molecules that are adsorbed on the surface of the nanoparticles, while the sustained release is due to the diffusion of the drug from the core of the nanoparticles. This release profile is desirable for wound healing applications, as the initial burst release can provide an immediate therapeutic effect, while the sustained release can maintain the therapeutic concentration of the drug for a prolonged period of time.

The release kinetics of both drugs were best described by the Korsmeyer-Peppas model, with a release exponent (n) of less than 0.5. This indicates that the release mechanism is primarily governed by Fickian diffusion, which is consistent with the release of drugs from a porous matrix (Katas et al. 2013, 2012; Gan and Wang 2007). The antibacterial activity of the nanoparticles was evaluated against *S. aureus*, which is a common pathogen found in wound infections. The DualCSNPs exhibited significant antibacterial activity, with an MIC of 2 mg/mL. This activity can be attributed to the synergistic effect of the encapsulated VCM and the intrinsic antibacterial properties of chitosan. The cytotoxicity study showed that the nanoparticles were biocompatible and did not cause any significant toxicity to HDFs at concentrations up to 4 mg/mL. This is an important prerequisite for any material that is intended for use in wound healing applications. The in vitro wound healing assay demonstrated that the EGF-containing nanoparticles significantly enhanced the migration of HDFs, which is a crucial step in the wound healing process. The wound closure rate was significantly higher in the cells treated with the EGFCSNPs and DualCSNPs compared to the control group. This indicates that the encapsulated EGF retains its biological activity and can effectively stimulate cell migration. The fact that the nanoparticle formulations were more effective than free EGF suggests that the nanoparticles may protect the growth factor from degradation and enhance its bioavailability (Mohammed et al. 2017).

It is noteworthy that the inclusion of VCM in the dual-loaded nanoparticles did not have any adverse effect on the cell migration-promoting activity of EGF. This is an important finding, as it suggests that the two drugs can be co-delivered without interfering with each other’s activity. The lack of a significant effect of VCM on cell migration is not surprising, as its primary role is to combat bacterial infection rather than to promote tissue regeneration. The combination of an antibacterial agent and a growth factor in a single formulation is a promising strategy for the treatment of infected wounds, as it can simultaneously address the two major challenges in wound healing: infection and impaired tissue repair (Costa and Sousa Lobo 2001; Contri et al. 2022).

This study has some limitations. The in vitro wound healing assay is a simplified model that does not fully recapitulate the complexity of the in vivo wound healing process. Therefore, further studies are needed to evaluate the efficacy of the DualCSNPs in an in vivo wound healing model. Additionally, the long-term biocompatibility and biodegradability of the nanoparticles need to be investigated. Future studies should also focus on optimizing the formulation to improve the encapsulation efficiency of VCM and to further control the release of the encapsulated drugs.

## Conclusion

In conclusion, we have successfully fabricated and characterized a dual-delivery system of chitosan nanoparticles co-encapsulating EGF and VCM. The nanoparticles exhibited desirable physicochemical properties, including a suitable particle size, a narrow size distribution, and a high positive zeta potential. The nanoparticles provided a sustained release of both drugs and were found to be biocompatible and effective in inhibiting the growth of *S. aureus*. Furthermore, the dual-loaded nanoparticles significantly enhanced cell migration in an in vitro wound healing assay. These findings suggest that the dual-loaded chitosan nanoparticles have the potential to be a promising therapeutic strategy for the treatment of infected wounds. However, further in vivo studies are needed to confirm their efficacy and safety.

## Data Availability

All source data for this work (or generated in this study) are available upon reasonable request.
